# Comparing catch-up vaccination programs based on analysis of 2012–13 rubella outbreak in Kawasaki City, Japan

**DOI:** 10.1371/journal.pone.0237312

**Published:** 2020-08-14

**Authors:** Chiyori T. Urabe, Gouhei Tanaka, Takahiro Oshima, Aya Maruyama, Takako Misaki, Nobuhiko Okabe, Kazuyuki Aihara

**Affiliations:** 1 Institute of Industrial Science, The University of Tokyo, Tokyo, Japan; 2 Graduate School of Engineering, The University of Tokyo, Tokyo, Japan; 3 Kawasaki City Institute for Public Health, Kawasaki, Japan; The Chinese University of Hong Kong, HONG KONG

## Abstract

During the 2012–13 rubella outbreak in Japan, local governments implemented subsidy programs for catch-up vaccination to mitigate the rubella outbreak and prevent congenital rubella syndrome (CRS). In most local governments, to prevent CRS, eligible persons of the subsidy program were women who were planning to have a child and men who were partners of pregnant women. On the other hand, in Kawasaki City, unimmunized men aged 23–39 years were additionally included in the eligible persons, because they were included in an unimmunized men group resulting from the historical transition of the national routine vaccination in Japan. The number of rubella cases in the city decreased earlier than that in the whole Japan. First, in order to estimate the effect of the catch-up vaccination campaign in Kawasaki City on the epidemic outcome, we performed numerical simulations with a Susceptible-Vaccinated-Exposed-Infectious-Recovered (SVEIR) model incorporating real data. The result indicated that the catch-up vaccination campaign showed a beneficial impact on the early decay of the rubella cases. Second, we numerically compared several different implementation strategies of catch-up vaccinations under a fixed amount of total vaccinations. As a result, we found that early and intensive vaccinations are vital for significant reduction in the number of rubella cases and CRS occurrences. Our study suggests that mathematical models with epidemiological and social data can contribute to identifying the most effective vaccination strategy.

## Introduction

Rubella remains as a worldwide endemic disease, except for the Americas, and its elimination is a crucial issue in public health all over the world [1, 2]. The most serious concern about rubella outbreaks is that rubella infection of a pregnant woman can cause congenital rubella syndrome (CRS) of the developing fetus, associated with miscarriage, foetal death, various severe anomalies including visual and hearing impairment, congenital heart disease, central nervous system damages, and growing retardation [2]. In particular, rubella infection during an early stage of pregnancy increases the risk of CRS. To prevent rubella infection and CRS, effective vaccination strategies have been studied [3]. The 2012–13 rubella outbreak in Japan (the total population: around 130 million) resulted in nearly 17,000 rubella cases and 45 CRS cases during 2012–14 [4–9]. The outbreak was mainly attributed to the infection of unimmunized men [5, 10–13]. In Kayano et al. [10], it was pointed out that a catch-up vaccination program targeting adult men could lead to a substantial reduction in the number of rubella cases, through an analysis with an epidemiological model based on renewal equations.

**Fig 1:**
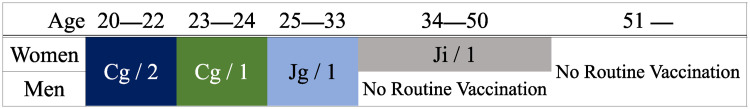
Transition of the national routine vaccination programs in Japan [10]. The numbers in the upper row indicate the ages of persons in April 2013. (Cg/2) Those who received the two-dose group vaccination when they were children. (Cg/1) Those who received the single-dose group vaccination when they were children. (Jg/1) Those who received the single-dose group vaccination when they were junior high school students. (Ji/1) Those who received the single-dose individual vaccination when they were junior high school students. Men aged over 34 years were unvaccinated in the national vaccination program.

In Japan, the national routine vaccination program launched in 1965. Fig 1 shows the transition of the national vaccination program, which is based on the surveillance report [11]. The numbers in the upper row indicate the ages of persons at April 1st, 2013, which is the beginning of the fiscal year (FY) 2013 in Japan. The middle and bottom rows show the type of the national routine vaccination for women and men, respectively. The labels indicate the condition when the persons received the routine vaccination. The top capital letters, C and J, represent Children (under seven years) and Junior high school students, respectively. The subsequent letters, g and i, represent group and individual vaccinations, respectively. In general, it is inferred that the vaccination coverage for the individual vaccination is smaller than that for the group vaccination. The last numbers, 1 and 2, after a slash indicates one-dose and two-dose vaccinations, respectively. Those aged over 23 years did not receive a two-dose vaccination, implying that they were not fully immunized. Thus, there were many susceptible persons in such age groups, especially in men. The unimmunized group seemed to be responsible for the rubella outbreak in Japan. Also in Romania and Poland, rubella outbreaks occurred under the immunization policies similar to that of Japan [14–16].

Following the rapidly growing number of reported rubella cases in the beginning of 2013 in Japan, local governments urgently started subsidy programs for promoting rubella vaccination. Eligible persons of the subsidy programs were selected by each local government, depending on the local epidemic situation and the fiscal condition, based on the guideline issued by the Ministry of Health, Labour and Welfare of the national government. In a notice released on 29th January 2013 from the Ministry, partners and families living with pregnant women, women aged from 15 to 39 years old, and women in an earlier puerperal period were recommended to receive vaccination to prevent CRS [17]. In most local governments, the eligible persons were women who were planning to have a child and men who were partners of pregnant women, since these two classes of persons were the top priority for reducing the risk of CRS. On the other hand, Kawasaki City and a few other local governments included unimmunized men aged 23–39 years in the eligible persons in addition to the above two classes by considering that they did not receive a double-dose group vaccination in the routine program as shown in Fig 1 [7]. Kawasaki City is one of the biggest cities in Japan and nearby the capital city, Tokyo (see S1 Fig). The special features of the program in Kawasaki City were to target the susceptible persons in the additional class and to implement the program early and intensively compared with many other local governments.

After the start of the catch-up vaccination, the number of rubella cases in Kawasaki City decreased earlier than that in the whole Japan [7], possibly due to the special catch-up vaccination program. However, the effect of the vaccination program on epidemic outcomes was not fully clarified. In this study, we aim to investigate the potential of strategic catch-up vaccination programs through numerical simulations of an epidemic mathematical model. To mimic the situation in Kawasaki City, we incorporated various relevant data into the model, including the weekly number of rubella cases, the population age distribution, the monthly vaccination data, the routine vaccination data, and the monthly number of birth in the city. We adopted a network model in which each individual is represented by a node and the contacts of individuals are represented by links between nodes. Each individual has an internal state categorized into susceptible, vaccinated, exposed, infectious, and recovered states. Individual-based simulations of the network model were performed to compare the epidemic outcomes under different vaccination strategies. The result indicated the effectiveness of the special vaccination strategy of Kawasaki City in a significant and early reduction of the number of rubella cases, although the epidemic size has considerable variations for simulation trials with different initial conditions [18]. Furthermore, we examined the effects of the initiation timing and the duration of the catch-up vaccination program on the epidemic outcomes under a fixed amount of total vaccinations. As a result, we found that not only an increase in the total amount of vaccination but also an early and intensive implementation of the catch-up vaccination program are indispensable for reducing the risk of rubella outbreaks and CRS.

## Materials and methods

### Rubella outbreak in Japan

**Fig 2:**
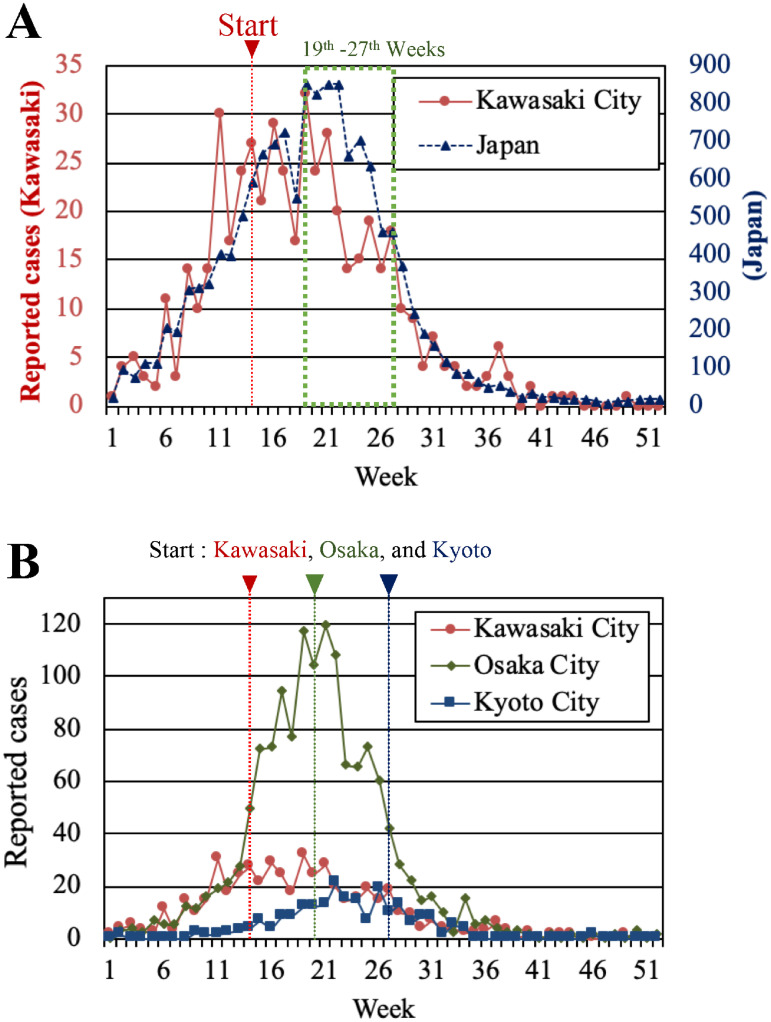
Comparison of 2013 rubella outbreaks between Kawasaki City, the whole Japan, and the two other cities, in 2013. (A) The numbers of weekly reported rubella cases in Kawasaki City (red circles with solid lines) and the whole Japan (blue triangles with dashed lines) [7, 19, 20]. (B) The numbers of weekly reported rubella cases in Kawasaki City, Osaka City (green diamonds with solid lines), and Kyoto City (blue squares with solid lines) [19, 21, 22].

We analyzed the data of reported cases in the FY2012–13 rubella outbreak in Japan. Fig 2A shows the number of weekly reported rubella cases in 2013 in Kawasaki City and the whole Japan [7, 19, 20]. Kawasaki City launched the subsidy program for the catch-up vaccination in the 14th week of 2013. After that, the number of rubella cases in Kawasaki City reached a peak at around the 19th week and then decreased earlier than that in the whole Japan. The decline of the rubella outbreak in the whole Japan was delayed several weeks from that in Kawasaki City. A possible cause of this salient gap is the difference of the catch-up vaccination programs between Kawasaki City and many other local governments.

For reference, Fig 2B compares the time course of the reported rubella cases in Kawasaki City (red circles) with those in the two other cities: Osaka City (green diamonds) and Kyoto City (blue squares). In the catch-up vaccination programs of both cities, the adult men who were not the partners of pregnant women were ineligible for the subsidies. Osaka City had approximately the same population density as Kawasaki City (see S1 Table for details). The catch-up vaccination campaign started on 13th May 2013 after the drastic increase in the rubella cases. Kyoto City had about one fifth of the population density of Kawasaki City (see S1 Table for details). The catch-up vaccination campaign started on 1st July 2013 after the number of rubella cases reached the peak. The number of cases per population in Kawasaki City is much smaller than that in Osaka City as recognized from S1 Fig and Fig 2B. In addition, the epidemic size in Kawasaki City is only slightly larger than that in Kyoto City despite the large gap in the population density. Therefore, we infer that the catch-up vaccination campaign in Kawasaki City was responsible for mitigating the epidemic size.

### Catch-up vaccination campaign

Since the catch-up vaccination campaign was started, totally 24,128 people were immunized by measles-rubella (MR) vaccines in Kawasaki City [7]. After excluding the data for 711 people whose vaccination coupons have defects, we obtained the information about the age, the gender, and the time of vaccination, for 23,417 people.

**Fig 3:**
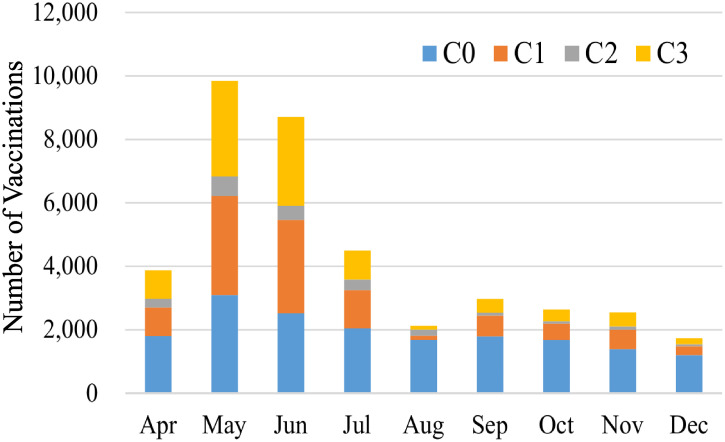
The monthly distribution of the number of vaccinations in Kawasaki City in 2013. The catch-up vaccination campaign launched on April 22nd, 2013. The vaccinations are divided into the following four classes: C0 (light blue), the routine vaccination; C1 (orange), the catch-up vaccination for women who were planning to have a child; C2 (gray), the catch-up vaccination for men who were partners of pregnant women; C3 (yellow), the catch-up vaccination for unimmunized men aged 23–39 years.

The monthly distribution of the number of vaccinations is shown in Fig 3. The vaccinations were categorized into the four classes:

- C0: the routine vaccination without catch-up vaccination.
- C1: the catch-up vaccination for women who were planning to have a child.
- C2: the catch-up vaccination for men who were partners of pregnant women.
- C3: the catch-up vaccination for unimmunized men aged 23–39 years.

During the catch-up vaccination campaign, the routine vaccination was continued.

We can observe a dramatic increase in the number of vaccinations in May and June 2013, which is obviously owing to the initiation of the catch-up vaccination program. The special features of the program in Kawasaki City were to target the susceptible persons in the additional class, which enabled an intensive vaccination during the early period, and to implement the program early compared with many other local governments. The subsequent decrease in the number of vaccinations on July was partly because C3 was tentatively excluded from the program due to the nationwide shortage of rubella-containing vaccines. The vaccinations except for C3 were continued during the catch-up vaccination campaign. The vaccinations in the first three months covered 62.6% of those in the entire period. The proportion of men in the vaccinated persons was around 50% in Kawasaki City, which was much higher than that in most other local cities. The addition of C3 to the target of the catch-up vaccination in Kawasaki City seems to have contributed to an increase in the proportion of men receiving vaccination. It also considerably increased the total number of vaccinations as shown in Fig 3.

### Mathematical model

**Fig 4:**
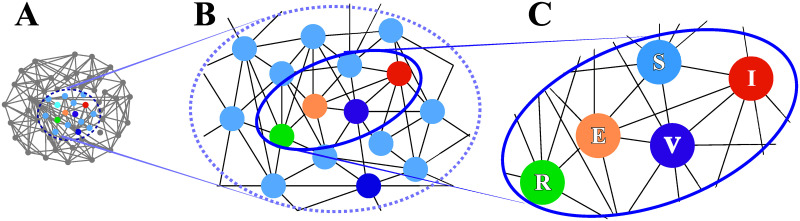
Schematic illustration of a contact network model for rubella epidemic processes. (A) A contact network of individuals. Individuals (nodes) have contacts with others (links between nodes). (B) An enlargement of a part of the network in (A). (C) Each individual has an internal state, S, V, E, I, or R, and the state changes with time (see Fig 5). Infection can occur through the links shared by infectious individuals.

Motivated by the special catch-up vaccination program in Kawasaki City, we investigated the effectiveness of several possible catch-up vaccination programs on the rubella outbreak, using a mathematical model. Our mathematical model describes rubella spreading on contact networks of individuals as illustrated in Fig 4A and 4B. We leveraged a network model, instead of a population model, in order to consider attributes of individuals, such as age and vaccination history. Additionally, through the attributes, we can incorporate various data in Kawasaki City into the model.

The network nodes correspond to individuals and the links represent contact relationships between individuals as shown in Fig 4. Each individual has an internal state which is susceptible (S), vaccinated (V), exposed (E), infectious (I), or recovered (R), as shown in Fig 4C. Infection of susceptible and vaccinated individuals can occur only through their links shared by infectious individuals.

**Fig 5:**
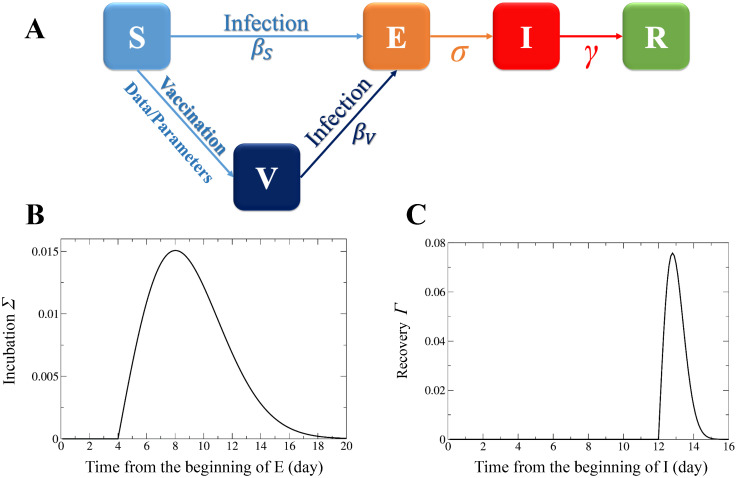
SVEIR model of rubella infection. (A) The transition diagram. The internal states of individuals are categorized into susceptible (S), vaccinated (V), exposed (E), infectious (I), or recovered (R) classes. The transmission rate from S to E is denoted by *β*_*S*_ and that from V to E by *β*_*V*_. The transition from S to V was assumed to occur according to the data on vaccination in Kawasaki City or with a transition probability parameter, depending on the simulation. (B) The expected value of the transition probability that an exposed individual changes to an infectious one at time *t* = *m*Δ*t*(*m* = 0, 1, 2, …). It is represented as $\Sigma \left(t\right)=\sigma \left(t\right)\Pi_{i=0}^{m-1}\left(1-\sigma \left(i\Delta t\right)\right)$ where the incubation rate *σ* in each time is is given by Eq (1). The parameter values were set at *a*_EI_ = 1.025, *b*_EI_ = ln(*a*_EI_)/4, and Δ*t* = 0.1 days. (C) The expected value of the transition probability that an infectious individual changes to a recovered one at time *t* = *m*Δ*t*(*m* = 0, 1, 2, …). It is represented as $\Gamma \left(t\right)=\gamma \left(t\right)\Pi_{i=0}^{m-1}\left(1-\gamma \left(i\Delta t\right)\right)$ where the recovery rate *γ* is given by Eq (2). The parameter values were set at *a*_IR_ = 6.5, *b*_IR_ = ln(*a*_IR_)/12, and Δ*t* = 0.1 days. See S1 Appendix for details about the settings of the parameters, *a*_EI_, *b*_EI_, *a*_IR_, and *b*_IR_.

The internal state of each individual makes transitions through events, such as vaccination, infection, disease progression, and recovery, as illustrated in Fig 5A. This model is called a Susceptible-Vaccinated-Exposed-Infectious-Recovered (SVEIR) model [23].

A susceptible individual transits to a vaccinated one by a vaccination. In numerical simulations, the amount of vaccination was determined from the real data in Kawasaki City or controlled as a system parameter. Newly vaccinated individuals were randomly chosen from susceptible individuals. In each week, an individual was randomly chosen one by one from susceptible individuals and then vaccinated. Then this procedure is repeated until the number of the newly vaccinated individuals reached the pre-determined number.

A susceptible individual can be changed by an infection to an exposed one, with transmission rate *β*_S_ per contact with an infectious individual. Considering a possibility of imperfect vaccination and a decay of immunization efficacy [12], we assumed that vaccinated individuals can also be infected with a very small transmission rate *β*_V_ (≪ *β*_S_) per contact with an infectious individual.

Each exposed individual transits to an infectious one with an incubation rate *σ*(*t*_E_), where *t*_E_ is the time elapsed from infection. Assuming typical exponential growth and decay of rubella viruses in the human body, we defined the incubation rate *σ*(*t*_E_) as follows:

$$\begin{array}{c} \sigma \left(t_{E}\right)=\{\begin{array}{cc} 1-a_{\text{EI}}\text{exp}\left({-b}_{\text{EI}}t_{E}\right) & (a_{\text{EI}}\text{exp}\left({-b}_{\text{EI}}t_{E}\right)<1),\\ 0 & (\text{otherwise}), \end{array} \end{array}$$ (1)

where *a*_EI_ and *b*_EI_ were assumed to be constant parameters. Because the incubation rate *σ*(*t*_E_) must be less than one, *a*_EI_ and *b*_EI_ have positive values. The length of the latent period (i.e. the period of an exposed state) of rubella is approximately nine days with range 4–15 days [24, 25]. To fit this epidemiological data, we adjusted the parameter values, as detailed in S1 Appendix.

Each infectious individual changes to a recovered one with a recovery rate *γ*(*t*_I_), where *t*_I_ is the time elapsed from the start of an infectious period. We defined the recovery rate *γ*(*t*_I_) in the same manner as *σ*(*t*_E_):

$$\begin{array}{c} \gamma \left(t_{I}\right)=\{\begin{array}{cc} 1-a_{\text{IR}}\text{exp}\left(-b_{\text{IR}}t_{I}\right) & (a_{\text{IR}}\text{exp}\left(-b_{\text{IR}}t_{I}\right)<1),\\ 0 & (\text{otherwise}), \end{array} \end{array}$$ (2)

where *a*_IR_ and *b*_IR_ were assumed to be positive constant parameters. The length of the infectious period of rubella is around 13 days [24]. The adjustment of the parameters is detailed in S1 Appendix.

### Data used for model simulation

We incorporated various data in Kawasaki City into the model simulation as summarized in Table 1. The initial states of the individuals were determined based on the age distribution, the vaccination history, and the antibody prevalence rate [26]. The catch-up vaccination in some simulations was assumed to be conducted based on that in Kawasaki City (see Fig 3).

**Table 1:** Data used for model simulation.

Variables and parameters	Data	Source
Age distribution, *N*_*a*_(0)	Age distribution of population*	Kawasaki City [27]
Initial vaccinated subpopulation, *V*_*a*_(0)	Vaccination history*	Kawasaki City & National report [28–30]
Initial recovered subpopulation, *R*_*a*_(0)	Antibody prevalence rate	National report [26]
Catch-up vaccinations, *V*_*a*_(*t*)	Catch-up vaccination campaign*	Kawasaki City
Monthly pregnancy rate *B*(*m*)	Monthly distribution of births*	Kawasaki City [31]
Age-specific pregnancy rate, *Q*(*a*)	Age distribution of pregnant women	National report [32]
Probability of CRS	Frequency of CRS occurrence	National report [33]

* Including data in Kawasaki City.

#### Age distribution of subpopulations

The initial internal states of individuals were determined based on age distributions of subpopulations in Kawasaki City. The age distributions in October 2013 are shown in S2A Fig. The total population of age *a* in Kawasaki City, $N_{a}^{K}$, is separated into subpopulations as follows:

$$\begin{array}{ccc} N_{a}^{K} & = & S_{a}^{K}+V_{a}^{K}+E_{a}^{K}+I_{a}^{K}+R_{a}^{K},\;\text{for}\;a=0,...,60 \end{array}$$ (3)

where $S_{a}^{K}$, $V_{a}^{K}$, $E_{a}^{K}$, $I_{a}^{K}$, and $R_{a}^{K}$ are the sizes of the susceptible, vaccinated, exposed, infectious, and recovered subpopulations of age *a* in Kawasaki City. The setting for the age range, 0 ≤ *a* ≤ 60, is described later.

We intended to estimate $S_{a}^{K}$, $V_{a}^{K}$, and $R_{a}^{K}$ under an assumption that E and I are absent before the initiation of the rubella outbreak. Since the data on the age-specific antibody prevalence rate in Kawasaki City was unavailable, we estimated the number of recovered subpopulations as follows. The size of subpopulation $R_{a}^{K}$ was assumed to be expressed as $R_{a}^{K}=A_{a}^{K}-V_{a}^{K}$ where $A_{a}^{K}$ is the size of age-specific immunized subpopulation, because a high level of antibody to rubella virus is caused by vaccination or recovery from rubella. First, $V_{a}^{K}$ was calculated from the record of the routine vaccination programs from 1977 to 2012 in Kawasaki City. It is noted that $V_{a}^{K}=0$ for *a* > 50 because there was no routine vaccination program before 1977. Second, $A_{a}^{K}$ was computed from the antibody prevalence rate [26]. Third, it was assumed that $E_{a}^{K}=I_{a}^{K}=0$ for all *a* before the initiation of the rubella outbreak. Finally, $S_{a}^{K}$ was obtained from Eq (3).

In model simulations, we set the initial states of individuals according to this age-specific proportion of the subpopulations.

#### Probability of CRS

To estimate the number of CRS cases in simulations, we considered pregnancy events for female individuals. The pregnancy probability *P*_prg_(*m*, *a*) that a female individual at age *a* gets pregnant on the *m*-th month (*m* = 1, 2, …, 12) in the year was assumed to be given by

$$P_{\text{prg}}\left(m,a\right)=cQ(a)B(m),$$ (4)

where *Q*(*a*) is the age distribution of pregnant women, *B*(*m*) is the monthly pregnancy rate, and *c* is a positive constant coefficient. According to the surveillance data [32], *Q*(*a*) was assumed to follow a Gaussian distribution with average 29.9 and standard deviation 4.3. For simplification, we assumed that a pregnancy occurs ten months before a birth, and calculated the probability of monthly pregnancy rate *B*(*m*) from the data of monthly birth rates in Kawasaki City. Parameter *c* controlling the scale of the number of pregnancies was set at *c* = 148 so as to be consistent with the number of births in the city.

In numerical simulations, the number of CRS cases was determined depending on the pregnancy probability for a female individual and the probability of CRS for a pregnant individual. We considered the fact that the risk of CRS is higher in the earlier stage of pregnancy. When a pregnant woman has apparent rubella infection, the frequency of CRS occurrence is 50% for 0–1 month pregnancy, 35% for 1–2 month pregnancy, 18% for 2–3 month pregnancy, and 8% for 3–4 month pregnancy, according to the national report [33]. In numerical simulations, we assumed that the probability of CRS for a pregnant individual follows the frequency of CRS occurrence.

### Simulation method

We simulated the network model of 100,000 individuals with initial internal states S and V, because recovered individuals at the initial time were not involved in the transmission dynamics. For simplicity, one half of the individuals in each age were assumed to be female and the remaining were to be male. This is because the actual male-to-female ratio of population in each age is close to one [34]. The gender was randomly assigned to each individual. The contact networks were given by random networks (Erdős–Rényi graphs) and fixed during a simulation run. A contact network was generated by assigning a contact relationship to each pair of individuals with probability *n*_c_/(*N* − 1). The distribution of the number of contacts per individual follows a bionomial distribution. Each individual was assumed to be in contact with *n*_c_ = 16 other individuals on average (see S1 Appendix for details). Consequently, as an initial condition, each individual has attributes; the age, the gender, and the internal state, S or V.

In simulations, we treated rubella infections among individuals aged 0–60 years including all persons who can receive vaccinations in the routine and the catch-up vaccination programs. Newborn individuals aged less than one year could receive vaccinations in the routine vaccination program. In the 2012–13 rubella outbreak in Japan, the number of infected population aged over 60 was very small [8]. Therefore, we did not consider such population in our simulation.

We investigated the time evolution of the sizes of susceptible, vaccinated, exposed, infectious, and recovered subpopulations of age *a* (*a* = 0, 1, …, 60) at time *t*, which are denoted by *S*_*a*_(*t*), *V*_*a*_(*t*), *E*_*a*_(*t*), *I*_*a*_(*t*), and *R*_*a*_(*t*). The total population, *N*(*t*), is expressed by the summation of the subpopulations as follows:

$$\begin{array}{ccc} N(t) & = & \sum_{a=0}^{60}\;(S_{a}\left(t\right)+V_{a}\left(t\right)+E_{a}\left(t\right)+I_{a}\left(t\right)+R_{a}\left(t\right)). \end{array}$$ (5)

**Fig 6:**
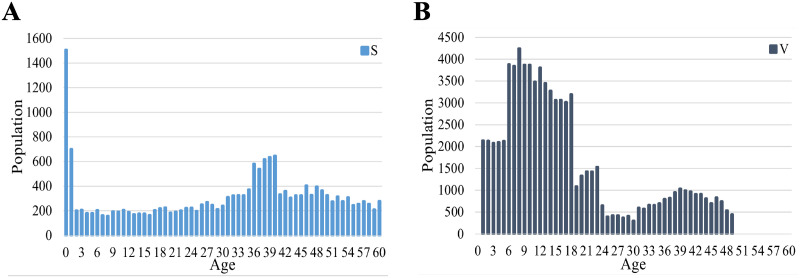
Age distributions of S and V subpopulations used in numerical simulations. (A) The estimated age distribution of the susceptible subpopulation. (B) The estimated age distribution of the vaccinated subpopulation.

The initial subpopulations in the age groups, *S*_*a*_(0), *V*_*a*_(0), and *R*_*a*_(0) (*a* = 0, …, 60), (see S2B Fig) were set to be in proportion to the estimated ones in Kawasaki City, $S_{a}^{K}$, $V_{a}^{K}$, and $R_{a}^{K}$ (*a* = 0, …, 60). The ratios of subpopulations, S, V, and R in Kawasaki City, $\Sigma_{a=0}^{60}S_{a}^{K}$, $\Sigma_{a=0}^{60}V_{a}^{K}$, and $\Sigma_{a=0}^{60}R_{a}^{K}$, were about 5%, 20%, and 76%, respectively. The initial subpopulations of S and V are shown in Fig 6. To trigger rubella transmission, ten susceptible individuals (*I*(0) = 10) were randomly chosen and changed to infectious ones (see S1 Appendix for details).

The time step for the simulation was set at Δ*t* = 0.1 (day) (see S1 Appendix for details). At every time step, a state transition of each individual can occur stochastically with the probabilities defined by Eqs (1) and (2). When Σ and Γ obtained from *σ* and *γ* are given as shown in Fig 5, the latent and infectious periods in the simulation were mostly less than 20 days and 16 days, respectively.

The amount of vaccinations was determined in proportion to the monthly catch-up vaccination in Kawasaki City or controlled as model parameters. At the beginning of every week, a given number of susceptible individuals were randomly chosen and changed to vaccinated ones. When a susceptible individual was linked with *k*_S_ infectious individuals, the effective transmission rate from S to E at time *t* was given by

$$\begin{array}{ccc} {\tilde{\beta }}_{S}\left(t\right) & = & 1-{\left(1-\beta_{S}\right)}^{k_{S}\left(t\right)}, \end{array}$$ (6)

Similarly, when a vaccinated individual was linked with *k*_V_ infectious individuals, the effective transmission rate from V to E was given by

$$\begin{array}{ccc} {\tilde{\beta }}_{V} & = & 1-{\left(1-\beta_{V}\right)}^{k_{V}\left(t\right)}. \end{array}$$ (7)

The transmission rates were set at *β*_S_ = 0.1 and *β*_V_ = 0.001 so that the scale of the rubella outbreak was similar to that of the actual one in Kawasaki City [7] (Fig 2) (see S1 Appendix for details). The pregnancy event was assigned randomly to female individuals according to the probability *P*_prg_(*m*, *a*).

The effectiveness of vaccination programs was evaluated in terms of the degree of reduction in the final epidemic size and the number of CRS cases. A simulation run was terminated when the number of infectious individuals became zero.

### Data sharing policy

All the data except for the catch-up vaccination campaign are publicly available [7, 19–22, 24–26, 28–33]. The data for the catch-up vaccination campaign were recorded by Kawasaki City from 22nd April 2013 to 31st March 2014. In this study, we used the data in from 22nd April 2013 to 31st December 2013. The catch-up vaccination campaign data are available upon requests to the Infectious Disease Control Measures Section, Public Health Center, Kawasaki City (contact address: 40kansen@city.kawasaki.jp).

### Ethical considerations

As for the catch-up vaccination campaign data, this study has been approved by the Ethics Committee of Kawasaki City Institute for Public Health (Number: 27-3). The ethics committee waived the requirement for informed consent.

## Results

We performed two types of simulations. The first one was for evaluation of the catch-up vaccination campaign in Kawasaki City. The amount of vaccinations was determined according to the real data of the routine vaccination C0 and the catch-up vaccination campaign including three classes C1–C3. Incorporating class C3 into the vaccination program is the main difference from the programs in many other local governments.

The second one was for seeking more effective implementation strategies of vaccination programs. In Kawasaki City, the total amount of vaccinations increased because of adding class C3. It is reasonable that the increase of vaccinations contributed to the reduction in the number of rubella cases. The other feature of the catch-up vaccination program was the early and intensive execution. We focused on the initiation timing and the length of catch-up vaccination program, and examined the dependency of these factors on the spread of rubella and the occurrence of CRS.

### Evaluation of the catch-up vaccination campaign

**Fig 7:**
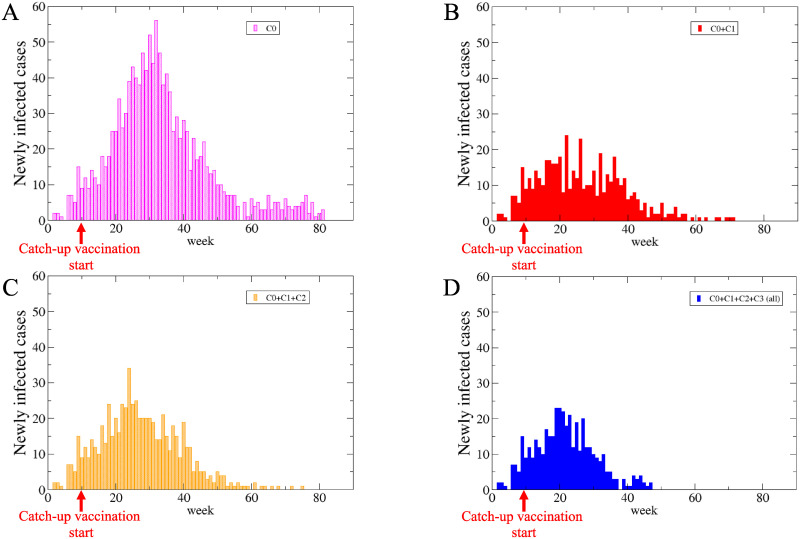
Newly infected cases under four different vaccination programs in simulation. Comparing vaccination programs: only routine program (A: C0), an addition of women who were planning to have a child (B: C0+C1), a further addition of men who were partners of pregnant women (C: C0+C1+C2), the catch-up campaign in Kawasaki City (D: C0+C1+C2+C3).

Fig 7 shows an example of the time course of the newly infected cases for different vaccination programs in the numerical simulation of the SVEIR model. The number of new infections was identical for all the vaccination programs until the catch-up campaign is started, because the initial condition, the network of individuals, and the pseudorandom number seed for probabilistic events were the same. In the vaccination program of C0 (Fig 7A), the peak was the highest and the duration of outbreak was the longest among the four programs. As the eligible persons increased, the epidemic size decreased; the colored area shrinks with adding classes C1 (Fig 7B), C2 (Fig 7C), and C3 (Fig 7D) one by one. The vaccination program of C0+C1+C2 (Fig 7C) corresponded to that conducted in most local governments. The vaccination program of C0+C1+C2+C3 (Fig 7D) corresponded to that in Kawasaki City and resulted in the smallest outbreak.

The result depends on the initial conditions and the realizations of the random contact networks (see S3 Fig for details).

### Comparison of different implementation strategies of catch-up vaccination programs

To seek a more effective implementation strategy of vaccination program, we compared multiple possible strategies based on simulations with different network realizations and initial conditions. The features of the catch-up vaccination campaign in Kawasaki City were the early and intensive execution as well as the increase in the amount of vaccinations by targeting adult men.

First, we changed the initiation timing and the length of the catch-up vaccination program as parameters: the vaccination program started at the *T*_s_th week and continued for *D* weeks. The total amount of vaccinations, *N*_V_, was fixed at *N*_V_ = 10, 000 to eliminate the effect related to the amount of vaccinations. This value corresponded to 2.6% coverage, meaning that about a half of initial S individuals were changed to V during a simulation run.

**Fig 8:**
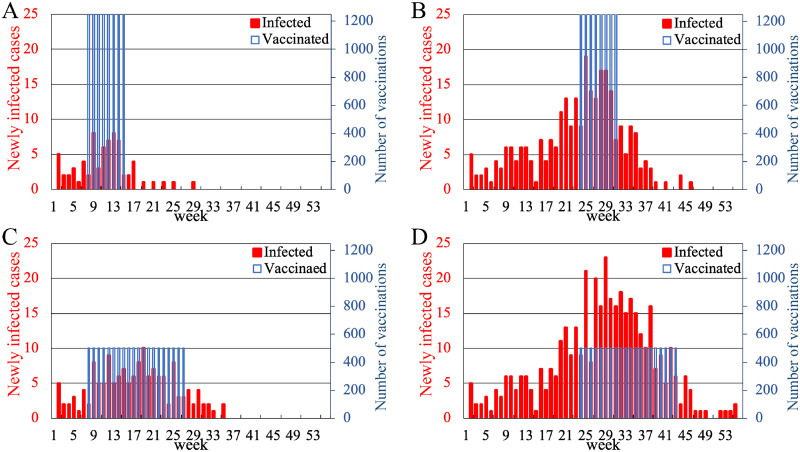
Dependence of the time course of newly infected individuals on the initiation timing and the length of catch-up vaccination program in simulations. The time course of the number of newly infected individuals (red bars) and the number of vaccinations (blue bars) in a simulation run. Figures (A)–(D) correspond to the cases with (*T*_s_, *D*) = (8, 8), (24, 8), (8, 20), and (24, 20), respectively. The total amount of vaccinations was fixed at 10,000.

Fig 8 shows typical time courses of the number of newly infected individuals and those of vaccinated individuals in each week. There are four implementation strategies regarding combinations of *T*_s_ = 8, 24 and *D* = 8, 20: (A) the early initiation of short-term intensive vaccination program, (B) the late initiation of short-term intensive vaccination program, (C) the early initiation of long-term distributed vaccination program, and (D) the late initiation of long-term distributed vaccination program. The numbers of vaccinations were spread uniformly during the period of the vaccination program, thus the number of weekly vaccinations was 1, 250 for *D* = 8 and 500 for *D* = 20 under the fixed total number of vaccination *N*_V_ = 10, 000.

For the early vaccination program as in Fig 8A (Fig 8C), the peak of the rubella outbreak was smaller than that for the late vaccination program as in Fig 8B (Fig 8D). For the short-term intensive vaccination program as in Fig 8A (Fig 8B), the number of newly infected cases decreased earlier than that for the long-term distributed vaccination program as in Fig 8C (Fig 8D).

The result indicates that the early and short-term vaccination program can most effectively decay the rubella outbreak as shown in Fig 8A. We infer that the early initiation of vaccination program swiftly reduced susceptible individuals before the outbreak reached the peak and resulted in the reduction in the peak. From the result on the intensity (the duration) of the catch-up vaccination program, we infer that, when the speed of an increase in the number of vaccinated individuals is faster than that of infection spread, the vaccination program can mitigate the spread of infection and decreases the newly infected cases.

The simulation results depend on the randomness of the contact network realizations, the initial conditions, and the stochasticity of the events. We statistically estimated the effect of early and intensive vaccination programs on a mitigation of the spread of infection. An outcome of a rubella outbreak was obtained through a simulation run for each combination of a network structure and an initial condition when the other parameter values were fixed. We obtained 50 outcomes using 10 different network realizations and 5 different initial conditions.

**Fig 9:**
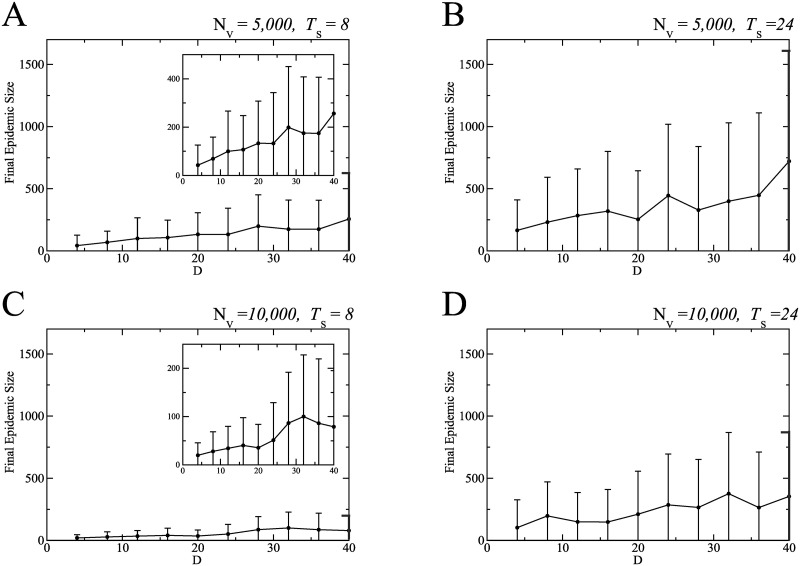
Final epidemic size in simulations. The final epidemic size depends on the amount of vaccinations, the initiation timing, and the length of vaccination program. Four panels correspond to different combinations of the vaccination programs and the implementation strategies: (A) the early initiation and the small amount of vaccinations, (B) the late initiation and the small amount of vaccinations, (C) the early initiation and the large amount of vaccinations, and (D) the late initiation and the large amount of vaccination. The horizontal axis indicates the duration of catch-up vaccination program, *D*.

Through a numerical simulation for each parameter set, we calculated the final epidemic size I_∞_ defined by the total population of infected individuals generated during the outbreak. In Fig 9, the filled circle and the error bar indicate the average final epidemic size and the standard deviation for the 50 outcomes, respectively. We considered two cases about the total number of vaccinations: the small amount case (*N*_V_ = 5, 000) as shown in the upper figures (Fig 9A and 9B) and the large amount case (*N*_V_ = 10, 000) as shown in the bottom figures (Fig 9C and 9D). The value *N*_V_ = 5, 000 means that a quarter of the initial S individuals were changed to V individuals during a simulation run. The left figures (Fig 9A and 9C) correspond to the early initiation of the vaccination program with *T*_s_ = 8 and the right figures (Fig 9B and 9D) to the late initiation with *T*_s_ = 24.

Fig 9 indicates that there are three important factors to mitigate the rubella outbreak: a sufficient amount of vaccinations (large *N*_V_), the earliness and intensiveness of vaccination program (small *T*_s_ and small *D*). The final epidemic size is decreased with an increase in *N*_V_. However, even if *N*_V_ is small, an increase in the epidemic size can be avoided by setting *T*_s_ at a small value. Even in the case with small *N*_V_ and large *T*_s_, the final epidemic size decreases with *D*. To sum up, when all the three factors reach sufficient levels, the catch-up vaccination program is most effective for mitigating the rubella outbreak. The mitigation is also achieved if at least one of the three factors is at a high level, even when the other two are not.

**Fig 10:**
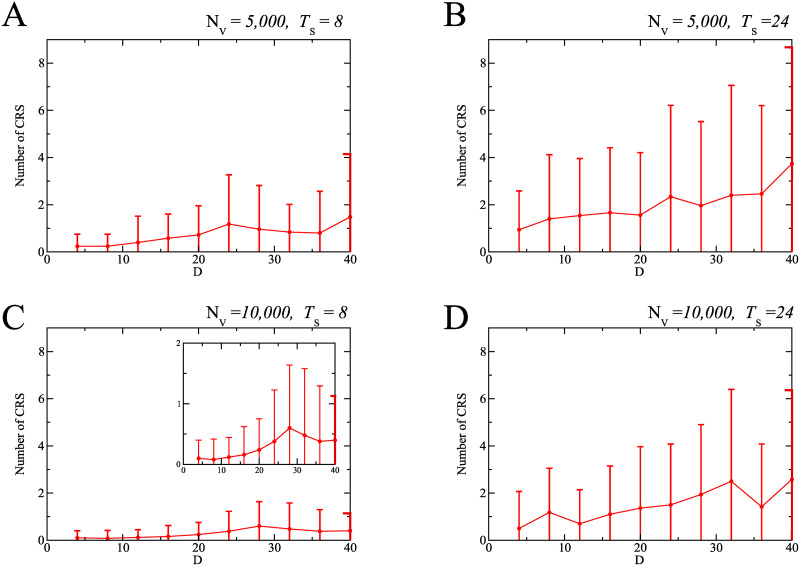
CRS occurrences in simulation. The number of CRS cases depends on the amount of vaccinations as well as the initiation timing and the duration of vaccination program. Four panels correspond to different combinations of the vaccination programs and the implementation strategies: (A) the early initiation and the small amount of vaccination, (B) the late initiation and the small amount of vaccination, (C) the early initiation and the large amount of vaccination, and (D) the late initiation and the large amount of vaccination. The horizontal axis indicates the duration of vaccination program, *D*.

The most serious concern in the rubella outbreak is CRS. Fig 10 shows CRS occurrences calculated based on the numerical simulations under the same condition as in Fig 9. The trend in Fig 10 is similar to that in Fig 9. The result shows that a sufficient amount of vaccinations under an early and intensive vaccination program could also be effective for reduction of CRS incidence.

CRS occurrences are related to rubella infection of female individuals in pregnancy and the CRS risk depends on the infection timing. We used various available data to estimate the number of CRS cases in simulations. The result was approximately consistent with the real data. In Japan, the number of reported rubella cases was 17, 000 and the number of reported CRS cases was 45 during 2012–14 [4–6, 8, 9]. The ratio of the number of CRS cases to that of rubella cases was about 0.3%. When the final epidemic size I_∞_ is minimum as shown in Figs 9C and 10C, the average values of I_∞_ and CRS cases were approximately less than 100 and 0.5, respectively. The ratio of the number of CRS cases to that of rubella cases is around 0.5%. When I_∞_ is maximum as shown in Figs 9B and 10B, the average values of I_∞_ and CRS cases were approximately less than 750 and 4, respectively. The ratio is also around 0.5%.

## Discussion and conclusions

Rubella outbreak occurred during 2012–13 in Japan. Local governments implemented catch-up vaccination campaigns in which eligible persons differ according to the situations of the local governments. In most local governments, eligible persons of the subsidy program were women who were planning to have a child and men who were partners of pregnant women. The main aim of the subsidy program was to prevent CRS occurrence. In Kawasaki City which is one of the biggest cities in Japan and located next to Tokyo, unimmunized men aged 23–39 years were additionally included in the eligible persons. There was a large group of the unimmunized men at that time due to the past national vaccination programs. The catch-up vaccination campaign in Kawasaki City aimed to promote vaccinations of people in such a group for mitigating the rubella infection and CRS occurrence. The rubella outbreak in Kawasaki City decayed earlier than that in most other cities and the whole Japan. However, this earlier decay was a single event and could be influenced by stochasticity. Therefore, it was not clear whether or not the earlier decay was due to the catch-up vaccination campaign.

To examine the early decay of rubella outbreak in Kawasaki City, we simulated a network-based mathematical model incorporating various real data to mimic the rubella outbreak in Kawasaki City. In the numerical simulations, the expansion of the eligible persons seemed to contribute to the reduction of rubella infections. We focused on the other feature of the catch-up vaccination campaign in Kawasaki City, that is the early and intensive implementation of the program. To investigate effective strategies in comparison with other possible strategies, we conducted numerical simulations with different initial conditions and network realizations. The result indicated that, when a sufficient amount of vaccination is provided and the catch-up campaign program is initiated early and intensively, the spread of rubella infection and the occurrence of CRS can be significantly mitigated by the catch-up vaccination program.

For preventing CRS, the top priority of the catch-up vaccination should be put on the high-risk targets, including women who are planning to have a child and partners of pregnant women. The immunization of other susceptible people is also indispensable for early mitigation of the outbreak. While the targets of the subsidy program for catch-up vaccination in Kawasaki City included unimmunized men, a large group of other unimmunized men still remains [7]. In the whole Japan, susceptible people aged around 30–40 years are also remaining [13, 35]. The enlightenment activity promoting vaccinations for the unimmunized people in these generations would be vital for elimination of rubella. A research suggested that additional initiatives for persons who do not believe that they directly benefit from vaccination is needed [36]. In Japan, most full-time workers can hardly find time to get vaccinations in week days [7]. Global and local governments need to remove barriers to vaccination through subsidy programs, corporate clinics, and holiday/night clinics.

The effective implementation strategy of a catch-up vaccination for rubella, such as early and intensive execution, would be applicable to other infectious diseases. In this study, the four combinations of the routine vaccination program (C0) and the catch-up vaccination program (C1-C3) were simulated. Other possible programs can also be tested through numerical simulations in a similar way. In our numerical simulations, various kinds of available real data were incorporated into the model to make it more realistic. We consider that models incorporating real data will be more important for better estimations of epidemic outcomes and designs of effective countermeasures.

In this paper, we discussed the catch-up immunization strategies for rubella in a short term in Japan. From a broader perspective, it would be desired to explore better additional and supplemental immunization strategies for other paediatric infectious disease such as measles through mathematical modeling. There are several studies on the supplemental immunization strategies using mathematical models [37–41]. A study on supplementary immunization activities in China indicates that adult-targeted supplemental immunization activities would become most effective in the long run [37]. In another study on vaccination strategies in New Zealand, additional vaccinations beyond routine childhood immunization were analyzed from a cost-benefit viewpoint in a long term to prevent measles outbreaks induced by imported viruses [39]. Even if rubella and measles are eliminated from a country, efforts to maintain a high-level immunization are required to prevent outbreaks induced by imported viruses [2].

Next we discuss the possibilities for improving our simulations. First, for simplification, the ratio of female and male population was set to be even in each age. Actually, the ratio in FY2013 was total female: total male = 0.967: 1.000. However, to estimate the effect of the vaccination program on CRS occurrences more accurately, the age-specific ratio should be reflected. In addition, we assumed a random contact pattern in the model. In reality the contact patterns could be more intricate and biased depending on the ages and genders. The contact pattens can influence on epidemic dynamics [42]. Due to the absence of human contact pattern data in Kawasaki City and in Japan, we assumed random contact networks in the present study. In the future, we would like to incorporate a realistic contact pattern estimated from real data into the mathematical model and its simulations.

Second, we estimated the recovered population using the age-specific antibody prevalence rate obtained from the national data, because such data in Kawasaki City was unavailable. If there was a difference in the antibody prevalence rate between Kawasaki City and the whole Japan, it could affect the estimation of the number of recovered population. In addition, even if those antibody prevalence rates were the same before the catch-up vaccination campaign, the antibody prevalence rate in Kawasaki City would differ from that in the whole Japan after the campaign. Therefore, if data for the age-specific antibody prevalence rate in Kawasaki City are available, that is desirable for simulation of rubella outbreak, especially for rubella outbreak after 2013.

Finally, we did not consider the population flow between Kawasaki City and the neighboring regions. In this study, we adopted static networks for representing the contact patterns of individuals. If we incorporate the population flow into simulation, we can use other model frameworks considering human mobility [18, 43, 44], although detailed data of mobility patterns are not yet available.

The above difficulties are related to availability of data. Data collection would become increasingly essential in the future. To examine what kind of data is useful and how various data can be utilized, systems incorporating various data as in our study are necessary. In the future, when various data will become available, we can implement simulations of more realistic outbreak and explore effective strategies to mitigate the outbreak. There are various potential countermeasures to mitigate the epidemic outbreak (e.g., vaccinations and closure of school classes). Countermeasures involve expenses and risks, and differ in the effectiveness, depending on the type of the infectious disease and the situations of the outbreak. Therefore, the countermeasures need to be carefully selected. We consider that mathematical models incorporating real data could provide a scientific validation to a selection of a specific countermeasure or a combination of countermeasures.

## Supporting information

S1 Appendix Parameter setting in the model simulations. (PDF)

S1 Fig Geographical location of Kawasaki City. (EPS)

S2 Fig Age distributions of actual populations in Kawasaki City and estimated subpopulations for the numerical simulation. (EPS)

S3 Fig Newly infected cases under the four different vaccination programs. The results of 50 simulation runs are superimposed in each panel. One sample is demonstrated in Fig 7.
(PDF)

S1 Table Population-related and vaccination-related information of Kawasaki, Osaka, and Kyoto Cities in FY2013. (PDF)
