# Supplementary material for: Comparing catch-up vaccination programs based on analysis of 2012–13 rubella outbreak in Kawasaki City, Japan
Source: PLoS One. 2020 Aug 14;15(8):e0237312. doi: 10.1371/journal.pone.0237312 (PMC7428070; doi:10.1371/journal.pone.0237312)
Supplement: S1 Appendix — (PDF) [file pone.0237312.s001.pdf]

Appendix: Parameter setting in the model simulations

Comparing catch-up vaccination programs based on analysis of 2012–13 rubella outbreak in Kawasaki City, Japan

Chiyori T. Urabe[1*], Gouhei Tanaka[1,2], Takahiro Oshima[3], Aya Maruyama[3], Takako Misaki[3], Nobuhiko Okabe[3], Kazuyuki Aihara[1]

1 Institute of Industrial Science, The University of Tokyo, Tokyo, Japan
2 Graduate School of Engineering, The University of Tokyo, Tokyo, Japan
3 Kawasaki City Institute for Public Health, Kawasaki, Japan

* chiyori@iis.u-tokyo.ac.jp

1 Parameters related to rubella

We assumed the incubation rate as a function of time $t_{\rm E}$ elapsed from infection as follows:

$$\sigma(t_{\rm E}) = \begin{cases} 1 - a_{\rm EI}\exp(-b_{\rm EI}t_{\rm E}) & (a_{\rm EI}\exp(-b_{\rm EI}t_{\rm E}) < 1), \\ 0 & (\text{otherwise}), \end{cases} \tag{1}$$

where $a_{\rm EI}$ and $b_{\rm EI}$ were assumed to be positive parameters.

The incubation period of rubella is normally around 17 days, ranging from 12 to 23 days, and viral shedding can start from seven days before rash onset [1, 2]. The latent period indicating the period without viral shedding is distinguished from the incubation period. The latent period ends at seven days before the last day of the incubation period as shown in Fig 1. Therefore, we assumed that the latent period is around 9 days, ranging from 4 to 15 days. In order to approximately reproduce this epidemiological information, the parameters, $a_{\rm EI}$ and $b_{\rm EI}$, of the incubation rate $\sigma(t_{\rm E})$ were set at $a_{\rm EI} = 1.025$ and $b_{\rm EI} = \ln(a_{\rm EI})/4$.

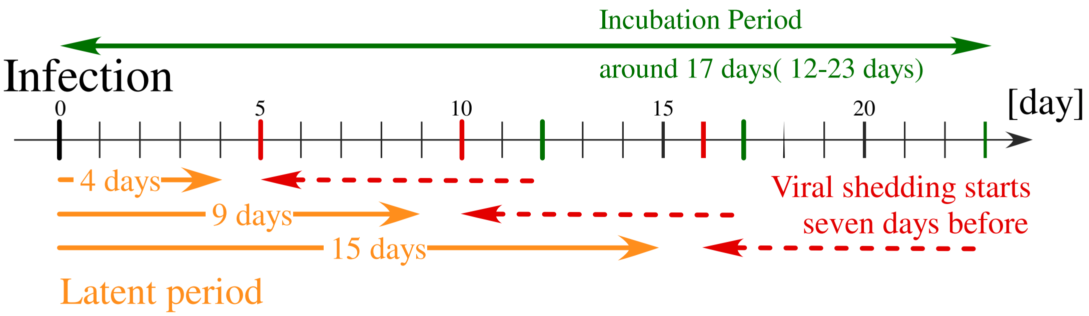

Fig 1. Estimation of the latent period. The incubation and latent periods are involved in the absence of symptom and the infectiousness, respectively.

We assumed the recovery rate as a function of time $t_{\rm I}$ elapsed from a change to I as follows:

$$\gamma(t_{\rm I}) = \begin{cases} 1 - a_{\rm IR}\exp(-b_{\rm IR}t_{\rm I}) & (a_{\rm IR}\exp(-b_{\rm IR}t_{\rm I}) < 1), \\ 0 & (\text{otherwise}), \end{cases} \tag{2}$$

where $a_{\rm IR}$ and $b_{\rm IR}$ are positiver parameters. Since the infection period of rubella is around 13 days [1], the parameters, $a_{\rm IR}$ and $b_{\rm IR}$, of the recovery rater $\gamma(t_{\rm I})$ were set at $a_{\rm IR} = 6.5$ and $b_{\rm IR} = \ln(a_{\rm IR})/12$.

2 The other parameters

In numerical simulations, infection events are related to the transmission rates, $\beta_{\rm S}$ and $\beta_{\rm V}$, the number of the initial infectious individuals, $I(0)$, the time step Δt, and the number of connection, $n_{\rm c}$. To mimic the rubella outbreak under the catch-up campaign in Kawasaki City, we determined the values through the following procedure.

First, as described above, the parameters determined from the property of rubella infection were fixed.

Second, we assumed the values of parameters except for n_c under restrictions; $0 < \beta_\mathrm{V}, \beta_\mathrm{S} < 1$, $\beta_\mathrm{V} \ll \beta_\mathrm{S}$, $I(0) > 0$, $I(0) \ll S(0)$, $\Delta t > 0$ [day], $\Delta t \ll 1$ [day]. We set as $\beta_\mathrm{S} = 0.1$, $\beta_\mathrm{V} = 0.001$, $I(0) = 10$, and $\Delta t = 0.1$[day].

Third, we adjusted the value of n_c, avoiding values for which no outbreak occurred through 50 simulation runs, and determined the value at $n_\mathrm{c} = 16$ to reproduce an outbreak which had approximately the same size of the rescaled rubella outbreak in Kawasaki City.

References

1. Centers for Disease Control and Prevention. Control and prevention of rubella: evaluation and management of suspected outbreaks, rubella in pregnant women, and surveillance for congenital rubella syndrome. MMWR Surveill Summ. 2001;50(RR12): 1-23. Available from: `https://www.cdc.gov/mmwr/preview/mmwrhtml/rr5012a1.htm`.

2. Centers for Disease Control and Prevention. Hamborsky J, Kroger A, Wolfe S, eds. Epidemiology and prevention of vaccine-preventable diseases. 13th ed. Washington D.C. Public Health Foundation; 2015. Available from: `https://www.cdc.gov/vaccines/pubs/pinkbook/index.html`.
