# Supplementary figures and images for: Comparing catch-up vaccination programs based on analysis of 2012–13 rubella outbreak in Kawasaki City, Japan
Source: PLoS One. 2020 Aug 14;15(8):e0237312. doi: 10.1371/journal.pone.0237312 (PMC7428070; doi:10.1371/journal.pone.0237312)

**A****C0**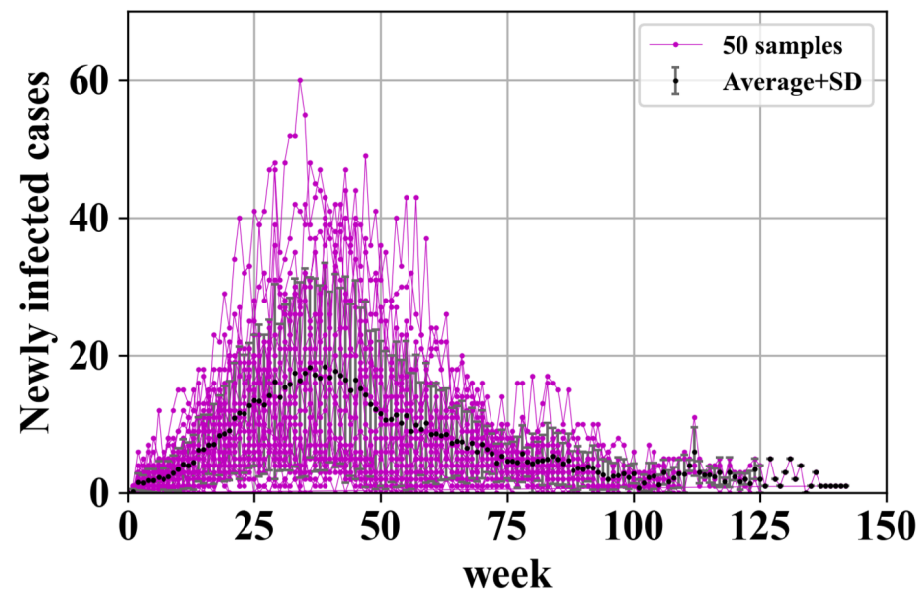**B****C0+C1**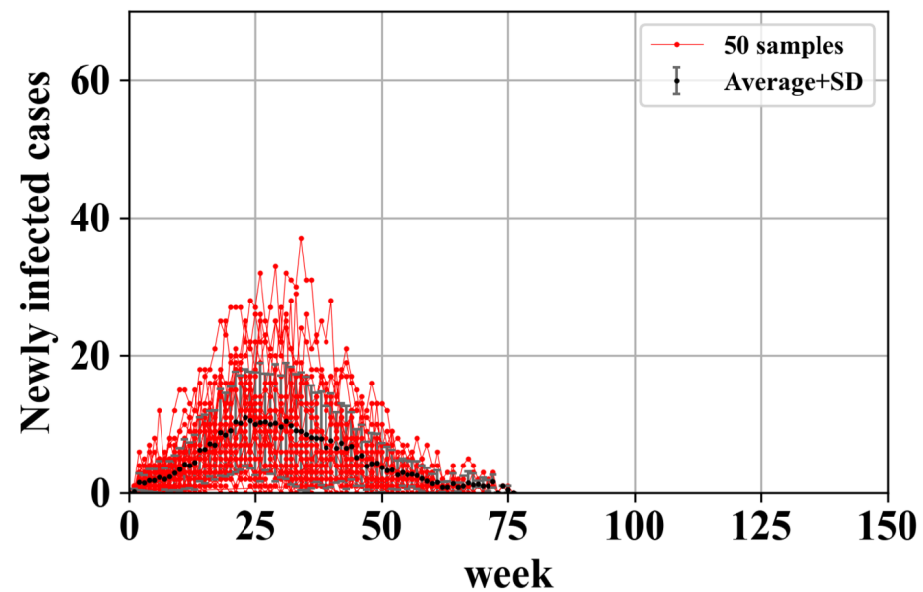**C****C0+C1+C2**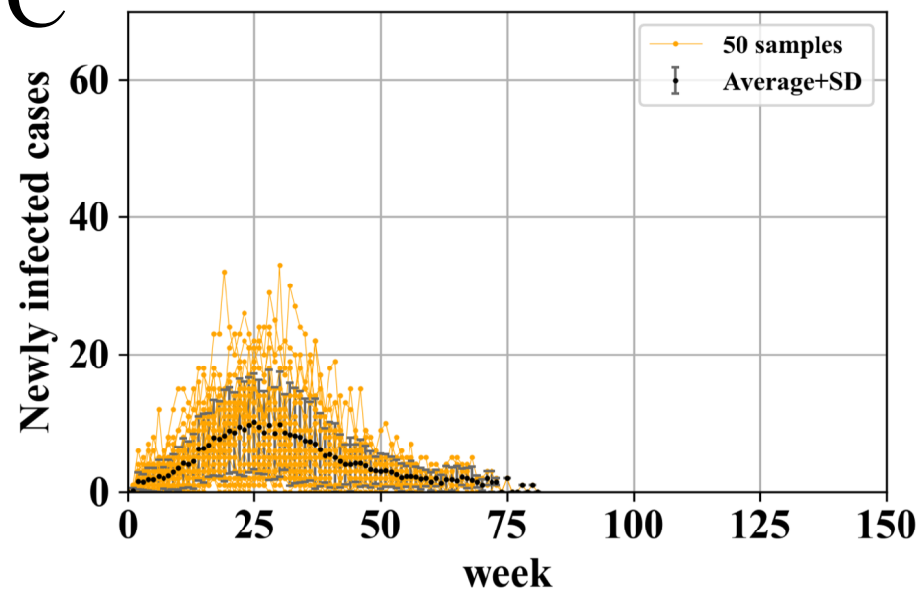**D****C0+C1+C2+C3 (all)**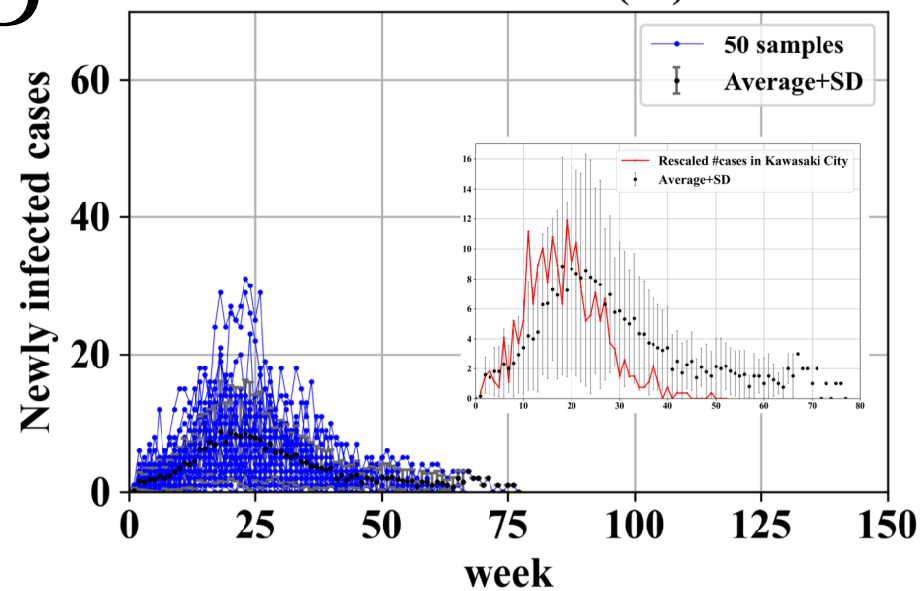

Supplement: S3 Fig — The results of 50 simulation runs are superimposed in each panel. One sample is demonstrated in Fig 7. (PDF) [file pone.0237312.s004.pdf]
