# Supplementary material for: Comparing catch-up vaccination programs based on analysis of 2012–13 rubella outbreak in Kawasaki City, Japan
Source: PLoS One. 2020 Aug 14;15(8):e0237312. doi: 10.1371/journal.pone.0237312 (PMC7428070; doi:10.1371/journal.pone.0237312)
Supplement: S1 Table — (PDF) [file pone.0237312.s005.pdf]

FY2013		Population & Density		Last accessed: 2nd December 2019.
**City**	**Population**	**Land Area [km^2]**	**Density [persons/km^2]**	**Source (in Japanease)**
Kawasaki	1,448,196	144.35	10,033	http://www.city.kawasaki.jp/shisei/category/51-4-3-1-1-0-0-0-0-0.html
				http://www.city.kawasaki.jp/170/page/0000009567.html
Osaka	2,683,487	225.30	11,911	https://www.city.osaka.lg.jp/toshikeikaku/page/0000203035.html
				https://www.city.osaka.lg.jp/toshikeikaku/page/0000402930.html
Kyoto	1,470,742	827.83	1,777	https://www2.city.kyoto.lg.jp/sogo/toukei/Population/Suikei/#t4
				https://www.city.kyoto.lg.jp/sogo/page/0000015581.html

FY2013	Eligible persons		DD/MM/YYYY	Last accessed: 17th December 2019.
**City**	**W & P**	**OM**	**Period**	**Source (in Japanese)**
Kawasaki	●	●	22/4/2013 - 31/3/2014	National Institute of Infectious Diseases. Infectious Agents Surverillance Report. 2016, Oct;37(10):20--22.
Osaka	●	×	13/5/2013 - 30/9/2013	http://warp.da.ndl.go.jp/info:ndljp/pid/8278401/www.city.osaka.lg.jp/kenko/page/0000219702.html
Kyoto	●	×	1/7/2013 - ?	https://healthnet.jp/wp-content/themes/main/pdf/paper/h2869.pdf
				https://www.city.kyoto.lg.jp/hokenfukushi/page/0000163145.html

● Eligible × Ineligible

W & P: Women who were planning to have a child and men who were partners of pregnant women

OM: Other adult men (e.g.. aged 23--39 years)
